# A scoping review of the impact of ageing on individual consumers' insurance purchase intentions

**DOI:** 10.1016/j.heliyon.2024.e37501

**Published:** 2024-09-07

**Authors:** Zhangwei Zheng, Hafizuddin-Syah B.A.M, Hafizah Omar Zaki, Qin Lingda Tan

**Affiliations:** Faculty of Economics and Management, Universiti Kebangsaan Malaysia, Malaysia

**Keywords:** Ageing, Insurance, Purchase intention, Young-old, Scoping review

## Abstract

Recently, the phenomenon of population ageing and its impact on the insurance industry has garnered increasing global attention. However, a notable gap in scholarly research persists in understanding the nuanced effects of ageing on consumer behaviour and insurance purchase intentions. This study maps the current academic evidence on how ageing influences individual consumers' insurance decisions. Using a scoping review methodology aligned with the Preferred Reporting Items for Systematic Reviews and Meta-Analysis extension for scoping reviews and Joanna Briggs Institute guidelines, 44 articles out of 1082 from four databases—Web of Science, Scopus, ScienceDirect, and Emerald Insight—are reviewed. The results reveal a rising interest in this research area, with China emerging as a significant contributor. The focus is predominantly on Theory of Planned Behavior, quantitative methods, questionnaire survey, regression analysis, older population, and general health insurance. Variables capturing the impact of ageing, beyond demographic information, include family-related, risk-related, and expectation-related factors. This study highlights the current state of research on ageing's effect on insurance purchase intentions and offers valuable insights and directions for future research.

## 1 Introduction

In recent years, population ageing has become a major concern for policymakers, insurers, and academics due to declining birth rates and increased life expectancy. According to definitions from the United Nations (UN) [1], the US Population Reference Bureau (PRB) [2], and the National Bureau of Statistics (NBS) of China [3], ageing is a dynamic process where the average age of a nation rises due to a growing proportion of older individuals or a shrinking proportion of younger people. According to UN standards, a society is classified as ageing, deep ageing, or super ageing when the percentage of individuals aged 65 or above is 7–14 %, 14–20 %, or above 20 %, respectively [1]. Currently, major global economies face ageing issues. For instance, China is becoming a deep ageing society, with 14.2 % of its population aged 65 and above in 2021 [3], equating to over 200 million people. This is a significant increase from just 7 % in 2000 [3]. Hence, China's rapid ageing highlights the importance of researching ageing and its impacts.

In consumer behaviour research, the impact of ageing has been extensively studied [[4], [5], [6], [7], [8], [9]]. However, there is limited research on how ageing affects insurance purchase intentions. As ageing progresses, consumers' purchasing behaviours and willingness to make purchases, especially regarding insurance, change significantly. Ageing influences not just individuals but also has broader societal implications, such as changes in labour markets, healthcare systems, intergenerational relationships, and social welfare [[10], [11], [12]]. For instance, high levels of ageing can lead to inadequate social benefits like pensions or delayed retirement [13], while low birth rates may impose future burdens on younger generations [14]. Ageing often alters risk perception, financial priorities, and outlook on the future [[15], [16], [17]], which can significantly affect how individuals engage with insurance services. Insurance, as a financial tool for security and risk mitigation, is crucial for protecting against unforeseen adversities [18]. Thus, exploring the impact of ageing on insurance purchase intentions is vital for both academic research and industry practice.

A search using relevant keywords reveals that no existing literature reviews have conducted a retrospective on this theme. Current reviews mainly focus on elderly consumer decision-making [[19], [20], [21]] or specific types of insurance [22], which do not align with this scoping review's scope. As the impact of ageing on insurance purchase intentions is emerging, this study uses a scoping review methodology to systematically assess the literature on this topic. The aim is to identify existing knowledge on how ageing influences insurance purchase intentions, map and summarise existing evidence, and highlight knowledge gaps to inform future research [23]. The central question is: How does ageing affect individual consumers' insurance purchase intentions? The specific sub-questions are: (1) What are the trends in publication years and study regions? (2) What are the theories and research methods used? (3) What types of insurance are covered? (4) What are the age characteristics of the subjects? (5) What variables and factors are used to measure the impact of ageing?

The subsequent structure of this article includes an introduction to the methods, a presentation of results based on the research questions, followed by a corresponding analysis and discussion. The final section provides conclusions and recommendations for future research.

## 2 Methods

### 2.1 Design

To understand the impact of ageing on insurance purchase intentions, this study employed a scoping review approach, which examines a wide range of literature sources and research designs to explore existing knowledge [23]. Scoping reviews are valuable for quickly identifying research trends and key concepts across diverse fields [24]. Following the guidelines of Norsworthy et al. [25] and Pham et al. [26], this review adhered to two main frameworks: the Preferred Reporting Items for Systematic Reviews and Meta-Analysis extension for scoping reviews (PRISMA-ScR) [27] and the Joanna Briggs Institute (JBI) guidelines [28]. The aim was to systematically and transparently explore how ageing affects insurance purchase intentions by gathering, reviewing, and synthesising relevant literature. Based on Arksey and O'Malley [24] and Peters et al. [28], the review followed key stages: defining research questions and objectives, searching for relevant studies, selecting studies according to inclusion and exclusion criteria, charting data (data extraction), and reporting results with conclusions and implications.

### 2.2 Search strategy

This study accessed existing literature using four online databases: Web of Science (WOS), Scopus, ScienceDirect, and Emerald Insight. The search strings (Table 1), derived from the research questions, included 'ageing', 'insurance', 'purchase', 'intention', and their synonyms. Given the novelty of the research topic and the lack of extensive prior studies, the search strategy imposed no time restrictions and did not limit results by language, ensuring a comprehensive and extensive literature compilation.

**Table 1:** Search strategies used in the scoping review.

Database	Search Strategy	Search results
Web of Science (WOS)	(((TS=(aging OR ageing OR senescence)) AND TS=(insurance)) AND TS=(purchas* OR buy* OR acquire OR obtain OR shopping OR procure OR decision-making)) AND TS=(intent* OR desire OR willingness OR motivation)	319
Scopus	(TITLE-ABS-KEY(aging OR ageing OR senescence) AND (insurance) AND (purchas* OR buy* OR acquire OR obtain OR shopping OR procure OR decision-making) AND (intent* OR desire OR willingness OR motivation))	512
ScienceDirect	(aging OR ageing) AND (insurance) AND (purchase OR buy OR decision-making) AND (motivation OR intention OR willingness)	34
Emerald Insight	abstract:'insurance' AND (abstract:'aging' OR 'ageing' OR 'senescence') AND (abstract:'purchase' OR 'purchasing' OR 'buying' OR 'buy' OR 'obtain' OR 'shopping' OR 'acquire' OR 'procure' OR 'decision-making') AND (abstract:'intention' OR 'intent' OR 'motivation' OR 'willingness' OR 'desire') + title:'insurance' AND (title:'aging' OR 'ageing' OR 'senescence') AND (title:'purchase' OR 'purchasing' OR 'buying' OR 'buy' OR 'obtain' OR 'shopping' OR 'acquire' OR 'procure' OR 'decision-making') AND (title:'intention' OR 'intent' OR 'motivation' OR 'willingness' OR 'desire')	217
Total	–	1082

### 2.3 Study selection

This review applied four inclusion and five exclusion criteria (Table 2). As detailed in Table 1, a total of 1082 papers were extracted from the four databases. After removing 71 duplicates using EndNote 20's de-duplication function, 1011 articles remained. Following the inclusion and exclusion criteria in Table 2, articles were screened by title and abstract, resulting in 927 irrelevant articles focusing on ageing economics, medical treatment of ageing-related diseases, and healthcare resource preferences. The full texts of 84 articles were reviewed, leading to the exclusion of 40 articles for failing to meet the criteria. Specifically, 10 articles were excluded due to unavailable original literature, one for being medical rather than business-related, and another for using only macro data. Additionally, 28 articles were excluded for not addressing both 'ageing' and 'insurance purchase intentions' together. Among these, 12 articles covered only ageing and not insurance purchasing intentions, focusing on topics such as retail banking, financial planning, and long-term care services. The remaining 16 articles addressed insurance purchase intentions but did not consider ageing, covering topics like agriculture insurance, flood insurance, and smart car insurance. It should be noted that the high number of excluded articles is typical due to the broad search scope, which includes titles, abstracts, and keywords, and requires manually removing many irrelevant articles due to the interdisciplinary nature of the research. Additionally, given that global ageing reached 7 % in 2007 (the threshold for an ageing society) [29], and that China—a major economic power and key contributor to ageing research—entered a deeply ageing society with over 14 % in 2021 [3], it is reasonable that this emerging topic has limited historical research.

**Table 2:** Inclusion and exclusion criteria for study selection.

Inclusion criteria	Exclusion criteria
1. Articles discussing the factors influencing 'insurance purchase intentions', which may involve age or ageing factors. 2. Articles discussing 'insurance purchase intentions' related to 'ageing', like 'ageing-related insurance', 'elderly consumers' insurance purchase intentions', or 'the impact of ageing on insurance consumption'. 3. Articles using microdata. 4. Articles focusing on individual consumers, rather than business consumers.	1. Articles for which the original full text cannot be found. 2. Articles that only focus on 'ageing' or 'insurance purchase intentions,' without any connection to the other topic. 3. Articles utilising only macrodata. 4. Articles that are conference abstracts, letters, research proposals, book chapters, or any other non-research paper. 5. Articles outside the field of business (e.g., management, marketing, finance), especially in the biological or medical fields.

**Fig. 1:**
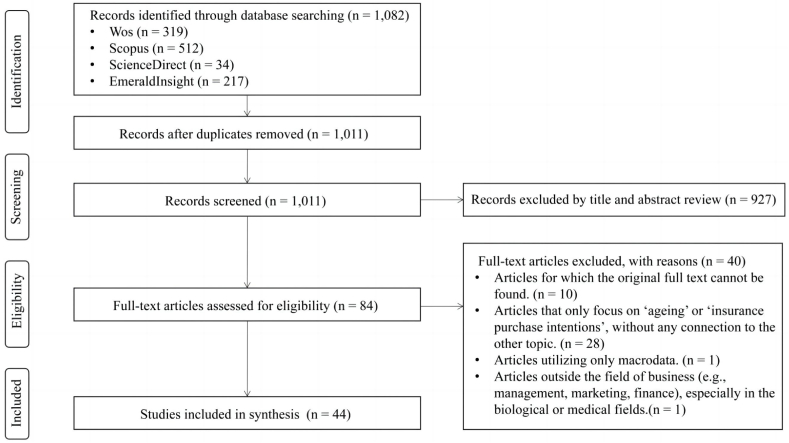
PRISMA-ScR flow diagram of studies search and selection.

During the selection process, four authors collectively established the inclusion and exclusion criteria. Two authors independently screened titles and abstracts based on these criteria. Discrepancies were resolved through discussions with the other two authors, preventing individual biases and errors. As this scoping review aims to map a broad spectrum of current evidence rather than critically appraise specific research contents, a quality assessment was not conducted. Hence, a total of 44 articles were finally selected for review. Fig. 1 illustrates the flow diagram of entire search strategy.

### 2.4 Data extraction and synthesis

Arksey and O'Malley [24] noted that a scoping review should determine the outcome of the research topic by extracting multiple key items. As a scoping review, it aims to map existing evidence on an emerging area. Therefore, four researchers developed a data-charting form based on PRISMA-ScR [27] and JBI [28] guidelines to determine the extraction items, facilitating data organisation from the articles. Specifically, items included the author, publication year, countries selected for research, types of insurance, research design, data collect and analysis method, number and characteristics of subjects (age groups), ageing-related factors, and findings. Two authors independently extracted relevant information from each article, while the other two reviewed the articles to verify data completeness and accuracy. The authors summarised findings aligned with the research question, using them as the foundation for the narrative synthesis.

## 3 Results

The search was conducted in September 2023. As shown in Fig. 1, a total of 1082 articles were obtained from four databases. After several rounds of screening, 44 articles were included in the review.

### 3.1 Publication years and countries

Table 3 presents the publication years and countries of the 44 studies. The earliest articles on the impact of ageing on insurance purchase intentions were from 2001, with no time limit set for this scoping review. The distribution is as follows: 2 articles (5 %) were published between 2001 and 2004, 5 (11 %) between 2005 and 2008, 3 (7 %) between 2009 and 2012, 12 (27 %) between 2013 and 2016, 9 (20 %) between 2017 and 2020, and 13 (30 %) between 2021 and 2023.

**Table 3:** Publication years and countries of the reviewed studies (n = 44).

Category	Description	N	%
Publication year	2001–2004	2	5 %
	2005–2008	5	11 %
	2009–2012	3	7 %
	2013–2016	12	27 %
	2017–2020	9	20 %
	2021–2023	13	30 %
Research countries	USA	13	30 %
	China	12	27 %
	Europe	2	5 %
	Spain	2	5 %
	Australia	2	5 %
	UAE	2	5 %
	Burkina Faso	1	2 %
	UK	1	2 %
	Italy	1	2 %
	Malaysia	1	2 %
	Iran	1	2 %
	Ghana	1	2 %
	Korea	1	2 %
	Pakistan	1	2 %
	Romania	1	2 %
	Slovak	1	2 %
	Multi-country	1	2 %

In terms of countries, the US and China (including Hong Kong, Macao, and Taiwan) were the most studied, representing 30 % and 27 % of the research, respectively. Europe, Spain, Australia, and the UAE followed, each with 5 %. Other countries, including Malaysia, the UK, Italy, Korea, Iran, Ghana, Pakistan, Romania, and Slovakia, were each represented once (2 %).

### 3.2 Theories and methods used

Table 4 outlines the theories and methods used in the 44 reviewed studies. The Theory of Planned Behaviour (TPB) was the most common theoretical framework, featured in 9 % of the studies. Utility Maximization Theory and the Theory of Intrafamily Moral Hazard each appeared in 7 % of the studies. Theories such as the Theory of Bounded Rationality, the Theory of Reasoned Action, and Insurance Demand Theory were used in 5 % of the studies, while Socioemotional Selectivity Theory and Rational Economic Theory were utilised in 2 % of the studies.

**Table 4:** Theories and methods used in the reviewed studies (n=44).

Discription	No.	%	Discription	No.	%
**Theory**			**Data analysis method**		
Theory of Planned Behaviour	4	9 %	Regression analysis (29)		
Utility maximization theory	3	7 %	*Logistic regression*	7	16 %
Theory of intrafamily moral hazard	2	5 %	*Multinomial logit regression*	3	7 %
Theory of bounded rationality	2	5 %	*Linear probability regression*	3	7 %
Theory of Reasoned Action	2	5 %	*Random-effects logistic regression*	2	5 %
Insurance demand theory	2	5 %	*Weighted multinomial regression*	1	2 %
Prospect theory	2	5 %	*Weighted logistic regression*	1	2 %
Socioemotional Selectivity Theory	1	2 %	*Two-limit tobit model*	1	2 %
Rational economic theory	1	2 %	*Single-equation probit regression*	1	2 %
State-dependent utility framework	1	2 %	*Probit regression*	1	2 %
Life course theory	1	2 %	*Ordered probit regression*	1	2 %
Commitment-trust theory	1	2 %	*Multivariate logistic regression*	1	2 %
Cue utilization theory	1	2 %	*Multinomial logistic regression*	1	2 %
Situational Theory of Problem Solving	1	2 %	*Linearized logistic regression*	1	2 %
Random Utility Theory	1	2 %	*Linear regression*	1	2 %
Theory of peer effects	1	2 %	*Conditional logit regression*	1	2 %
Expected utility theory	1	2 %	*Bivariate probit regression*	1	2 %
Trust transfer theory	1	2 %	*Binary logit regression*	1	2 %
Fuzzy Trace Theory	1	2 %	*Binary logistic regression*	1	2 %
Unified Theory of Acceptance and Use of Technology (UTAUT)	1	2 %	Structural equation modeling (6)		
			*SEM*	3	7 %
**Research design**			*PLS-SEM*	3	7 %
quantitative	38	86 %	Contingent Valuation Method (6)		
qualitative	4	9 %	*CVM (bidding game)*	3	7 %
mix	2	5 %	*CVM (double-bounded dichotomous choice)*	1	2 %
			*CVM (take-it-or-leave-it)*	1	2 %
**Data collection method**			*CVM (single-bounded discrete referendum)*	1	2 %
Questionnaire	23	52 %	Correlation analysis (3)		
Secondary data	17	39 %	*Correlation analysis*	2	5 %
Focus group	3	7 %	*Spearman's correlation test*	1	2 %
Experiment	2	5 %	Discrete choice experiment	3	7 %
Systematic review	1	2 %	Content analysis	3	7 %
			Difference-in-Differences analysis	1	2 %
			Double hurdle model	1	2 %
			Neural network	1	2 %
			Microsimulation approach	1	2 %
			Repeated measures analysis	1	2 %

For research design, quantitative methods were dominant, employed in 86 % of the studies, with qualitative methods used in 9 % and mixed methods in 5 %. Data collection methods predominantly involved questionnaires (52 %) and secondary data (39 %). Focus groups and experiments were less common, used in 7 % and 5 % of the studies, respectively, while systematic reviews appeared in 2 % of the studies. Regarding data analysis methods, regression analysis was the most prevalent data analysis method, used in 29 studies. Logistic regression, the most frequently applied regression technique, appeared in 16 % of the studies. Multinomial logit regression and linear probability regression were used in 7 % of the studies each. Other regression techniques included random-effects logistic regression and weighted multinomial regression. Structural equation modelling (SEM and PLS-SEM) was used in 7 % of the studies, and the contingent valuation method (CVM) in various forms was also employed in 7 % of the studies.

### 3.3 Types of insurance covered

This scoping review extracted insurance types directly from the original articles with minor adjustments for categorisation and analysis. For instance, 'private medical insurance' and 'private medical plans' were combined as 'medical insurance,' while 'longevity insurance,' 'annuity insurance,' and 'longevity annuities' were classified as 'longevity annuity insurance.' 'Takaful' and 'Islamic insurance' were standardised as 'Islamic insurance (Takaful).'

Table 5 lists the insurance types covered in the 44 reviewed articles. The total number of insurance types exceeds 44 (46) because two articles addressed multiple types. Health insurance (30 %) and long-term care insurance (28 %) were the most frequently mentioned, followed by life insurance and medical insurance (11 % each), Islamic insurance (Takaful) and longevity annuity insurance (7 % each), travel insurance (4 %), and social pension insurance (2 %).

**Table 5:** Types of insurance covered in the review (n=46).

Insurance	N	%
health insurance	14	30 %
long-term care insurance	13	28 %
life insurance	5	11 %
medical insurance	5	11 %
Islamic insurance (Takaful)	3	7 %
longevity annuity insurance	3	7 %
travel insurance	2	4 %
social old-age insurance	1	2 %

### 3.4 Age characteristics of respondents/participants

Given that ageing is most directly reflected in the increasing proportion of the older population, this study extracted age characteristics of respondents/participants from the articles to map the target groups. Articles not specifying the age profile were assumed to cover an age range from 18 to 100 years, excluding minors. Of the 44 articles, 43 considered age characteristics, as one systematic review did not address the study population.

Table 6 displays the age characteristics of the study populations in the 43 articles. This study uses the UN classification to define individuals aged 65 and above as the older population and those below 65 as the young-old population. Further categorisation follows the Organisation for Economic Co-operation and Development (OECD) standards [30]: individuals aged 16–24 are young adults, 25–54 are prime working-age adults, and 55–64 are older adults. This dual reference to UN and OECD criteria enhances the detail and scope of demographic analysis. Table 6 shows that 50 % of the studies focused on the older population, followed by prime working-age adults (32 %), older adults (13 %), and young adults (5 %).

**Table 6:** Age characteristics of respondents/participants in the review (n = 43).

Age	Description	%
16–24	Young adults (young-old)	5 %
25–54	Prime working-age adults (young-old)	32 %
55–64	Older adults (young-old)	13 %
65–100	Oldest old	50 %

**Fig. 2:**
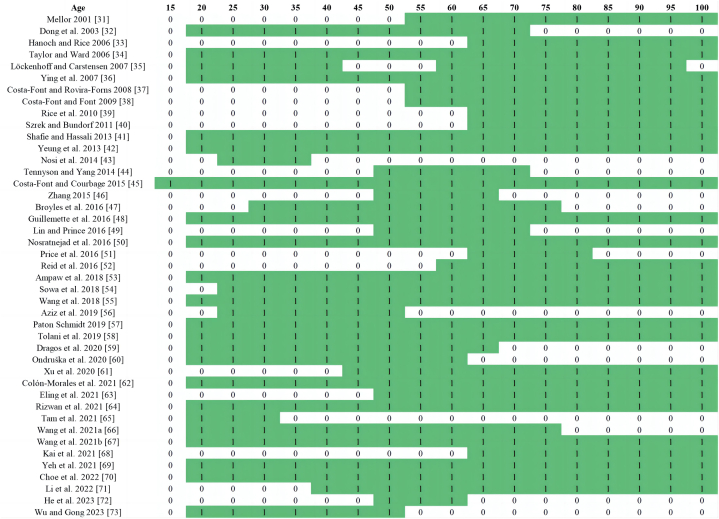
Age distribution of respondents/participants in the review.

Fig. 2 shows the specific age groups covered in each study and their overlap [[31], [32], [33], [34], [35], [36], [37], [38], [39], [40], [41], [42], [43], [44], [45], [46], [47], [48], [49], [50], [51], [52], [53], [54], [55], [56], [57], [58], [59], [60], [61], [62], [63], [64], [65], [66], [67], [68], [69], [70], [71], [72], [73]]. The green square represents age groups addressed in the article (denoted by 1), while the white areas indicate those not covered (denoted by 0). This figure supports the findings in Table 6, highlighting that most studies focused on the older population. Among the young-old population, studies on prime working-age adults were most common, followed by those on older adults and young adults.

### 3.5 Influencing factors related to ageing

This study extracted variables capturing the impact of ageing from the 44 articles and classified them into five categories: demographic, family-related, risk-related, cognitive-related, and expectation-related. Table 7 presents these results. To streamline categorisation and analysis, similar variables were combined. For instance, 'family size,' 'number of children,' and 'number of older individuals' were grouped as 'number of dependents.' Concepts related to 'perceived likelihood of needing care in the future' or 'perceived inability to live independently' were categorised as 'anticipated dependence.' 'Cognitive decline' and 'cognitive ability (numeracy and cognitive reflection)' were combined into 'cognitive abilities.' Variables such as 'presence of a chronic disease,' 'number of chronic diseases,' and 'perceived health conditions' were merged as 'health status.' 'Risk aversion' and 'risk attitudes' were classified as 'risk propensity.' Additionally, 'homeownership (which leads to a tendency to self-insure)' and 'accessibility of family care' were grouped as 'family insurance expectations (self-insurance).'

**Table 7:** Influencing factors related to ageing in the review (n = 44).

Category	Ageing related variables	N	%
Demographic information	Age	44	100 %
	Marital status	23	52 %
	Health status	23	52 %
	Number of dependents	17	39 %
	Saving behaviour	4	9 %
	Presence of private insurance coverage	2	5 %
Family-related	Interpersonal influence	5	11 %
	Caregiving experience	2	5 %
	Bequest motive	2	5 %
Risk-related	Risk perception	7	16 %
	Risk propensity	3	7 %
Cognitive-related	Cognitive abilities	5	11 %
	Cognitive age	1	2 %
Expectation-related	Family insurance expectations(self-insurance)	5	11 %
	Anticipated dependence	4	9 %
	Life expectancy expectations	2	5 %
	Public insurance expectations	2	5 %
	Perceived economic instability	1	2 %

Table 7 reveals that most studies used demographic information to assess the impact of ageing. Specifically, age was used in all studies, while 52 % included marital status and health status. The number of dependents was considered in 39 % of studies, savings behaviour in 9 %, and private insurance coverage in 5 %. Family-related variables were less common, with 11 % of studies examining interpersonal influence, and 5 % each investigating caregiving experience and bequest motives. Risk-related factors were used by 16 % of studies for risk perception and 7 % for risk propensity. Cognitive-related factors were discussed in 11 % of studies for cognitive ability and 2 % for cognitive age. Expectation-related factors included family insurance expectations (self-insurance) in 11 % of studies, anticipated dependence in 9 %, and life expectancy and public insurance expectations in 5 % each. An additional 2 % referenced perceived economic instability, reflecting concerns about the future economic security of respondents' adult children.

## 4 Discussion

**Fig. 3:**
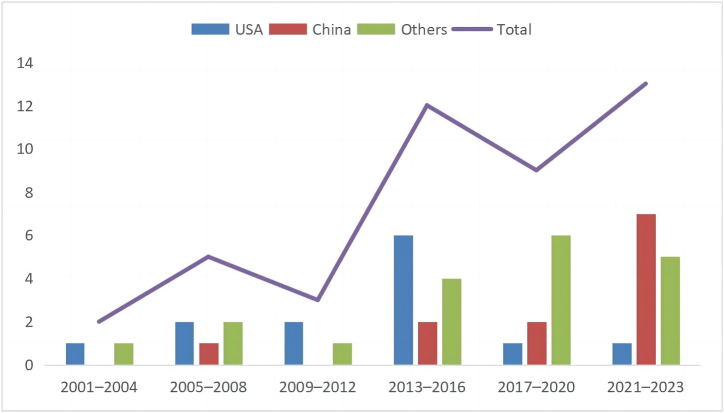
Number and trend of publications per years (by country).

The limited number of publications (44 articles over 23 years) suggests that this research topic is a niche within the broader field of ageing. Despite fluctuations, Fig. 3 shows a consistent increase in studies on the impact of ageing on consumers' insurance purchase intentions. The peak in publications occurred between 2021 and October 2023, indicating a rapidly growing trend, with future publications expected to rise further. This rise aligns with the global trend of population ageing. The UN [29] reports that 10 % of the global population was aged 65 or older in 2022, with projections estimating that this figure will reach 16 % by 2050. This demographic shift is driving increased research interest. Recent studies also highlight a surge in research across ageing-related fields, reflecting a growing academic focus on ageing's implications [74]. The increase in publications mirrors these demographic changes and indicates a rising global concern about the impact of ageing on various aspects, including insurance purchase behaviour. Thus, this growing research area is timely and relevant, addressing the challenges of ageing populations, and will become increasingly significant in the future.

Regarding research countries, the US has the highest number of published articles (13, or 30 % of the total). However, temporal patterns reveal a different trend (Fig. 3). From 2001 to 2016, the US saw an increase in publications, peaking between 2013 and 2016 with six articles. However, from 2017 to 2023, publications dropped significantly to just two. This decline can be attributed to several factors. The US's ageing population increased from 13.89 % in 2013 to 14.67 % in 2016 [75], surpassing the UN's deep ageing society threshold of 14 % [1]. This focus on ageing issues led to a temporary rise in research. However, as the US exceeded the 7 % ageing threshold well before 1960 [29], research on ageing was long established. Consequently, the transition to deep ageing may have only briefly intensified interest, with attention shifting to new issues as the immediate impact faded. In contrast, China published five articles with a steady trend from 2001 to 2020. From 2021 to 2023, publications surged to seven, making China the second-highest contributor with 12 articles (27 %). This surge coincides with China reaching a deeply ageing society threshold of 14.2 % in 2021 [3]. China crossed the 7 % ageing threshold only in 2000 and rapidly reached 14 % by 2021 [3]. This swift transition suggests that ageing research in China is still developing. With a population over four times larger than the US and unique ageing issues [76], research interest in China is likely to grow for many years, unlike the US's swift decline. Thus, the increased focus on ageing in China underscores its growing importance as a research region in the coming years.

The dominance of the TPB and Utility Maximization Theory reflects a reliance on established behavioural and economic theories to examine insurance purchase intentions in the context of ageing. The frequent use of multiple regression techniques highlights efforts to understand complex relationships and predict outcomes based on various factors. The emphasis on quantitative methods reveals a preference for empirical analysis and statistical validation. Although qualitative and mixed methods are less common, they suggest an interest in exploring context-specific aspects of insurance behaviour. The range of data collection methods, from questionnaires to experiments, demonstrates a multifaceted approach. The prevalence of questionnaires and secondary data indicates a focus on broad-scale analysis, while experiments and focus groups are used to explore specific behaviours and opinions. The limited use of systematic reviews points to the relatively early stage of comprehensive literature reviews in this field.

For the types of insurance covered in the studies, as shown in Table 5, except for social old-age insurance (2 %), which belongs to social insurance, all other types of insurance fall under the broader concept of commercial life insurance (as distinguished from property insurance) [18]. For instance, long-term care insurance (28 %) and medical insurance (11 %) are types of health insurance. When combined with the 30 % of studies focused on health insurance, a total of 69 % of research is centred on the broader category of health insurance. This is understandable, as the health problems and medical risks associated with ageing are most relevant to health insurance. However, given that broad insurance categories have been widely studied, future research should target specific subcategories such as medical insurance, long-term care insurance, and longevity annuity insurance, while reducing focus on general categories like life and health insurance.

In terms of age characteristics, as evident from Fig. 2 and Table 6, research trends rank the age of study subjects from high to low as older adults, prime working-age adults, and young adults. Notably, this analysis may contain inaccuracies as many articles do not specify the age ranges of study subjects. In this review, such articles (13) were assumed to cover ages 18–100 years, resulting in a high proportion of prime working-age categories. If age 65 years is used as the dividing line between ageing and non-ageing populations [1], only 8 of the 44 articles specify that their subjects are below 64 years [43,46,56,59,60,65,72,73]. Thus, only 18 % of studies on the impact of ageing on insurance purchase intentions focus on non-ageing populations, and most studies concentrate on older adults. Therefore, future research should emphasise the influence of ageing on the insurance purchase intentions of prime working-age populations.

The factors associated with ageing were also analysed. Table 7 shows that four variables (age, marital status, health status, and number of dependents) account for over 30 % of the evidence, all within the category of demographic information. This prevalence is due to the widespread collection of such data in empirical studies. However, these factors do not fully capture the multifaceted impact of ageing. Since this review aims to understand the impact of population ageing on individual consumers rather than intrinsic changes as they age, cognitive-related factors are not considered reflective of the 'ageing impact' in this study.

The remaining three categories—family-related, risk-related, and expectation-related variables—capture the nuanced impact of ageing more effectively than demographic information. For example, the variable of interpersonal influence reflects societal pressure from ageing through interactions with peers, older parents, and adult children [43,47,61,68,73]. The variable of caregiving experience indicates whether an individual has a history of providing care, offering insight into their understanding of ageing's challenges [44,47]. By contrast, 'bequest motivation' measures the older demographic's willingness to purchase insurance to increase their estate's inheritance [49,72]. Risk-related variables, such as risk perception [37,38,45,58,68,70,73] and risk propensity [48,63,69], assess an individual's judgement of risks associated with ageing, including their inclination to purchase insurance. Expectation-related variables illustrate ageing's impact on individual consumers. Family insurance expectations (self-insurance) reflect an individual's estimation of self-insurance feasibility and their attitude towards purchasing insurance [31,37,45,69,72]. Anticipated dependence measures pessimism about future independence, influencing the need for commercial insurance [45,61,72,73]. Public insurance expectations gauge confidence in public coverage reliability [45,50]. Life expectancy expectations estimate the extent of old-age risks [45,63], while perceived economic instability relates to expectations for future economic development and personal income under the influence of ageing [47]. These variables collectively evaluate ageing's impact on the willingness to purchase insurance.

The findings of this study have significant implications for policymakers, industry practitioners, and consumers. Policymakers must develop inclusive health insurance policies that cater to the 'young-old' demographic, integrating flexible coverage options, preventive care, and chronic disease management, while considering affordability and accessibility. These policies should prioritise comprehensive care over acute interventions. Industry practitioners can leverage these insights to design and market insurance products that address the specific needs and preferences of consumers under ageing context, such as combining health insurance with wellness programmes and personalised health plans. This research empowers consumers to make informed decisions regarding health insurance by understanding the importance of selecting products that offer comprehensive coverage tailored to their evolving needs. Overall, the study enhances the understanding of ageing's influence on insurance decision-making, aiding in the development of effective, consumer-centric health insurance policies and products.

## 5 Future directions

There is an increasing interest in how ageing affects consumer insurance purchase intentions, as evidenced by the continual surge in the number of articles on this topic. This indicates that it is an emerging research area warranting further exploration. Therefore, based on the aforementioned analysis, this study proposes future research directions from five perspectives.

In terms of geographical contribution, China is steadily cementing its position as a pivotal contributor in global ageing studies and is anticipated to remain a significant research hotspot in the coming years. This underscores the imperative for future research on this topic to either focus on China or at least reference studies from this region. Moreover, future research could also delve deeper into regional variations, examining how cultural and socioeconomic differences influence consumer behaviour in insurance markets under ageing.

For theories, future research should expand theoretical frameworks by incorporating variables such as risk perception, expectations, and family dynamics into established theories like the Theory of Planned Behavior (TPB) and Utility Maximization Theory. This integration could provide a more nuanced understanding of insurance purchase intentions in the context of ageing. Additionally, for methods, moving beyond purely quantitative methods to include mixed methods approaches will offer richer insights into the contextual factors influencing insurance decisions. Integrating cutting-edge technologies, such as blockchain [77] and deep learning models [78], may assist in uncovering more nuanced insights into consumer behaviour. Furthermore, conducting systematic reviews is also crucial to identify existing gaps in the literature and guide future research efforts more effectively.

Concerning the types of insurance examined, there exists a significant focus on general health insurance, highlighting a research gap. Future studies are thus encouraged to investigate more specific insurance categories within the context of ageing, in order to reveal more detailed insights. For instance, with the introduction and growing emphasis on the United Nations Sustainable Development Goals, comprehensive health coverage has become an objective for many countries. Consequently, the exploration of various health insurance subcategories, such as community-based health insurance, village-based health insurance, government-funded health insurance, and inclusive health insurance, is warranted, particularly in relation to specific contexts.

As to the age characteristics of study subjects in ageing studies, the literature is predominantly centred on the older population, with a dearth of studies involving the 'young-old' population. Considering the extensive societal impact of ageing, which permeates all age groups, the young-old population—a cornerstone of the consumer base—warrants increased scholarly focus in future investigations. Investigating the insurance purchase intentions of younger demographics could provide valuable insights into how these groups are preparing for ageing.

Regarding the variables employed to assess the ageing impact, while numerous studies have incorporated demographic data such as age, this approach alone is inadequate for fully encapsulating the influence of the ageing phenomenon. In contrast, variables pertaining to family dynamics (e.g. interpersonal influence), risk factors (e.g. risk perception) and expectations (e.g. anticipated dependence) are more apt for reflecting the societal impact of ageing on individuals. Future research could also explore other potential variables that may reflect the impact of ageing, such as imitation and information elements [79]. The interplay between these aforementioned variables, within the context of ageing, and consumers' insurance purchase intentions presents a promising research trajectory for future studies.

In conclusion, this study suggests the following future research directions: (1) How do cultural and socioeconomic differences between countries like the USA and China affect individual insurance purchase intentions under ageing? (2) How do qualitative or mixed methods approaches reveal the impact of ageing on insurance decisions? (3) What factors influence consumer decision-making for specific insurance types such as community-based health insurance under ageing? (4) How do insurance purchase intentions and behaviours differ between young-old population and older population in response to ageing? (5) How do factors such as interpersonal influence, risk perception, and anticipated dependence affect consumers’ insurance decision-making under ageing?

## 6 Conclusion

This scoping review comprehensively examined how ageing impacts insurance purchase intentions, identifying key research gaps and future directions. Out of 1082 articles from four major databases (WOS, Scopus, ScienceDirect, and Emerald Insight), 44 were reviewed. The growing scholarly interest underscores the importance of this emerging topic. The analysis reveals a strong reliance on established theories like the TPB, with a dominance of quantitative methods and regression analyses, alongside an emerging use of qualitative and mixed methods, and a need for more systematic reviews. The findings show a significant focus on general health insurance, highlighting the need for research into various insurance subcategories. The study emphasises the need to explore ageing across different cultures and socio-economic contexts, particularly in countries like the USA and China. It also calls for more focus on the 'young-old' demographic and other underrepresented age groups. Furthermore, expanding the analysis to include family dynamics, risk perceptions, and expectations will provide deeper insights into ageing and consumer behaviour in the insurance sector, offering valuable guidance for policy development and market strategies.

This study faced a limitation in analysing age characteristics as some referenced studies did not specify age ranges, necessitating the assumption that participants spanned the entire adult age spectrum, excluding minors. This assumption may lead to an over-representation of younger individuals within the older population, potentially affecting statistical accuracy. Future research should analyse study content to specify target age groups, such as older or young-old. Additionally, due to the limited number of articles, this study did not perform a quality assessment based on JCR quartile rankings. Given the scoping review's broad evidence mapping goal, future research could undertake a systematic review including quality assessment to better explore the current state and gaps in this field.

## Ethics approval statement

Review and/or approval by an ethics committee and consent by participants/respondents were not needed for this study because this is a scoping review article and no empirical data was gathered in the course of this research.

## Conflict of interest disclosure

The authors have no competing interests to declare that are relevant to the content of this article.

## Funding statement

The authors did not receive support from any organization for the submitted work.

## Data availability statement

Data sharing is not applicable to this review article since there was no generation or analysis of new data in the study.

## CRediT authorship contribution statement

**Zhangwei Zheng:** Writing – review & editing, Writing – original draft, Visualization, Methodology, Formal analysis, Data curation, Conceptualization. **Hafizuddin-Syah B.A.M:** Writing – review & editing, Supervision, Methodology, Formal analysis, Conceptualization. **Hafizah Omar Zaki:** Writing – review & editing, Supervision, Methodology, Formal analysis, Conceptualization. **Qin Lingda Tan:** Writing – review & editing, Methodology, Formal analysis, Data curation, Conceptualization.

## Declaration of competing interest

The authors have no competing interests to declare that are relevant to the content of this article.
